# Response to the letter to the editor: Metabolic comorbidities and post-transplant outcomes in Metabolic dysfunction-Associated Steatotic Liver Disease (MASLD): a cohort study from a Brazilian tertiary center

**DOI:** 10.20945/2359-4292-2026-0024

**Published:** 2026-03-19

**Authors:** Felipe Ramos Caprini, Fernanda Fernandes de Souza, Ajith Kumar Sankarankutty, Roberta Chaves Araújo

**Affiliations:** 1 Clínica Médica, Hospital das Clínicas, Faculdade de Medicina de Ribeirão Preto, Universidade de São Paulo, Ribeirão Preto, SP, Brasil; 2 Divisão de Gastroenterologia, Departamento de Clínica Médica, Faculdade de Medicina de Ribeirão Preto, Universidade de São Paulo, Ribeirão Preto, SP, Brasil; 3 Divisão de Cirurgia Digestiva, Departamento de Cirurgia e Anatomia, Faculdade de Medicina de Ribeirão Preto, Universidade de São Paulo, Ribeirão Preto, SP, Brasil

Dear Editor,

We thank the authors for their thoughtful Letter to the Editor ^(1)^ regarding our article on metabolic comorbidities and post-transplant outcomes in MASLD ^(2)^. We appreciate the constructive methodological considerations and address them below:

**1. Regarding immortal time bias in MASLD classification:** We recognize the potential impact of immortal time bias, a known challenge in studies that define exposures or outcomes dependent on a survival pe-riod. It is important to note that, given the retrospective nature of our study and the reliance on medical record data collected between 2005 and 2015, a rigorous reclassification of MASLD as a time-dependent exposure with the granularity necessary to mitigate this bias entirely was not feasible. At our institution, post-transplant clinical follow-up to investigate possible injuries to the transplanted liver is performed using liver biopsy or imaging studies, depending on the clinical indication and availability. In real-world practice, patients do not always attend on the scheduled date or can undergo the requested tests at the desired frequency. Therefore, it was not possible to obtain a continuous, standardized MASLD diagnostic status for patients throughout post-transplant follow-up, a prerequisite for such a time-dependent exposure analysis.

**2. Stratification by fibrosis stage:** In this retrospective study, systematic data on post-transplant fibrosis stratification were not available in medical records. We used available data in accordance with routine clinical practice. Additionally, the five-year follow-up period would be insufficient to document significant fibrosis progression in grafts, particularly given the natural history of MASLD. We acknowledge, however, that systematic fibrosis assessment would be valuable in future prospective studies with extended follow-up and standardized evaluation protocols.

**3. Pre-transplant diagnostic methods for MASLD:** We fully agree that histological assessment represents the gold standard for MASLD diagnosis. In our retrospective study, we used data from medical records, which, although potentially resulting in misclassification bias, reflect routine clinical practice in many liver transplant centres. Future prospective studies with standardized diagnostic protocols, including systematic biopsies, would provide more robust data on this issue.

**4. Regarding adjustment for immunosuppressive therapy:** The influence of immunosuppressive therapy on post-transplant metabolic outcomes is undeniable and was acknowledged in our Discussion. However, obtaining detailed, time-varying data on immunosuppressive regimens (type, dosage, adjustments, adherence) across 15 years of follow-up poses significant challenges regarding data completeness and accuracy. Importantly, our observational, retrospective study aimed to characterize the clinical-epidemiological profile of MASLD patients undergoing transplantation and describe associations with outcomes, rather than establish causal relationships. We acknowledge that correlation does not imply causality, and findings should be interpreted as descriptive associations. Future prospective studies should meticulously capture and adjust for these confounding effects.

In conclusion, we express our sincere gratitude for the thoughtful and constructive methodological considerations that will undoubtedly improve future research in this field. We appreciate these observations and recognize our study’s limitations. Future prospective research with standardized methods will enhance the understanding of MASLD transplant outcomes.

## Data Availability

the datasets used and/or analyzed during the current study available from the corresponding author on reasonable request.
